# The prevalence and severity of late postnatal anaemia amongst women at the Buea and Limbe Regional Hospitals in Cameroon

**DOI:** 10.11604/pamj.2025.51.41.40494

**Published:** 2025-06-11

**Authors:** Nancy Labu Nji, Nicholas Tendongfor, Roussel Ngetsche Ambebe, Julie Nchung Ashu, Magouanet Sandrine Tchio, Jules Clement Assob Nguedia, Gregory Edie Halle-Ekane

**Affiliations:** 1 Nkwen Baptist Hospital, Cameroon Baptist Convention Health Services, Bamenda, Cameroon,; 2 Department of Public Health and Hygiene, Faculty of Health Sciences, University of Buea, Buea, Cameroon,; 3 Martin Luther King Junior Memorial Foundation, Buea, Cameroon,; 4 Department of Obstetrics and Gynecology, Faculty of Health Sciences, University of Buea, Buea, Cameroon,; 5 Bonaberi Baptist Hospital, Cameroon Baptist Convention Health Services, Douala, Cameroon,; 6 Department of Biological Sciences, Faculty of Medicine and Pharmaceutical Sciences, University of Douala, Douala, Cameroon

**Keywords:** Late postnatal anaemia, prevalence, severity, Cameroon

## Abstract

**Introduction:**

late postnatal anaemia is not routinely investigated in lower-middle and low-income countries. Hence, it remains undiagnosed and untreated. There are quality of life, morbidity, and mortality implications of anaemia in this period. The objective of this study was to determine the prevalence, severity, and factors predisposing to late postnatal anaemia amongst women in the Buea Regional Hospital (BRH) and the Limbe Regional Hospital (LRH), Cameroon.

**Methods:**

a cross-sectional study amongst 355 women between the 6^th^ to 27^th^ week postpartum was conducted. Data collection using a structured questionnaire and capillary hemoglobin (Hb) was measured with the Sejoy® Hemoglobinometer. Late postnatal anaemia was defined by a Hb < 12g/dl between the 6^th^ to 27^th^ week postpartum. The prevalence of anaemia was calculated as the proportion of women with a haemoglobin level less than 12g/dl in the LPNP concerning the total population of women studied. The severity of anaemia was categorized into mild, moderate, and severe. The relationship between late postnatal anaemia (LPNA) and predictor variables was analyzed by the binary logistic regression method for odds ratios and p-values. Data analysis using SPSS version 23 with statistical significance set at a p-value < 0.05 at a 95% confidence interval.

**Results:**

there were 109 women out of the 355 participants with anemia, giving a prevalence of 30.7%. A majority of whom had mild and moderate anemia, that is 61 (56%) and 46 (43.1%) respectively, with only 1 (0.9%) participant with severe anemia. Level of education (p= 0.030), trimester of commencing antenatal care consultations (p=0.001), prenatal anemia (p=0.003), the baby??s birth weight (p= 0.047), and mucocutaneous pallor (p<0.001) were significantly and independently associated with late postnatal anemia.

**Conclusion:**

late postnatal anaemia is relatively common in this study setting, demonstrating a prevalence of 3 in 10 women affected. However, most cases are with mild to moderate anaemia. Determinants found were a low level of education, late commencement of antenatal care, prenatal anaemia, and mothers with low-birth-weight babies, plus mucocutaneous palor.

## Introduction

The postnatal period (PNP), also known as the postpartum or puerperal period, refers to the days and weeks following the delivery of the baby and is characterized by the physiological return of the woman to her pre-pregnant state [1]. The postnatal period has been defined from delivery till the onset of menses or following delivery till the end of lactation, which is usually from 8 weeks to 32 weeks following delivery [2]. The postnatal period is divided into three phases, namely the acute phase which is the first 24 hours following the delivery of the placenta, the early phase, which is after 24 hours to 7 days following delivery and the late phase which is from the 8^th^ day to 6 weeks or 6 months [3]. Although an essential time in obstetrics, the PNP is the most neglected. Less focus is given to the mother as the attention of both the family and healthcare workers is tilted towards the neonate [4]. This neglect is further demonstrated in the relatively small number of studies looking at this period compared to the antenatal period, especially regarding anaemia [5].

Anaemia can be defined as either a decrease in circulating red blood cells (RBC) or erythrocytes, a decrease in haemoglobin concentration, or a decrease in haematocrit from its normal [6]. This definition varies with age, sex, altitude, smoking status, and physiological status like pregnancy [7]. Postnatal Anaemia (PNA) has been defined as a haemoglobin (Hb) level less than 11g/dl or 12g/dl at the first or eighth week postpartum, respectively [2]

During pregnancy, the plasma volume expands by about 50% from the 6^th^week [8], meanwhile red blood cells increase only by about 18-25%; as such, it results in a physiologic anaemia or haemodilution anaemia [9]. The hypervolemia enables women to lose about 30% of blood volume with minimal change in the hematocrit value at delivery [2]. Following delivery, the resolution of the haemodilution leads to a rise in haemoglobin. With a blood loss of < 300 ml, the hemoglobin increases to the level before delivery [10]. With the combination of the postpartum haemorrhage and haemodynamic changes, the postpartum Hb is allowed to stabilize after delivery before a diagnosis of anaemia can be made. This is usually by the 48^th^ hour following delivery. But this diagnosis is most reliable by 1 week postpartum [2]. Some studies have proven that iron deficiency typically resolves between the 4^th^ to 6^th^ week postpartum, in low-income women, it lasts longer [11].

The causes of anaemia in the postnatal period are similar to those in the pregnant state. However, the relative anaemia of pregnancy, which accounts for most of the iron deficiency anaemia in pregnancy, ceases to exist in the PNP. Nonetheless, infectious causes like malaria and other systemic infections amongst other aetiologies such as nutritional deficiencies (e.g folate, B12 deficiency), haemoglobinopathies and red cell enzymopathies persist in the PNP [12]. The severity of LPNA can be divided into mild, moderate, and severe as per the haemoglobin value. Mild anemia is defined as a haemoglobin level between 11.0 to 11.9g/dl, moderate anemia between 8 and 10.9 g/dl and severe anemia as Hb less than 8g/dl [7].

The prevalence of anaemia when greater than 40% is a severe public health problem [6]. Worldwide, the prevalence of postnatal anaemia is high (41.8%) and affects women in both high, middle-, and low-income countries. In 2017, a study carried out in Yaoundé, Cameroon the prevalence of early postpartum anemia was found to be close to the worldwide prevalence (46.6%) [13]. However, there is an overall paucity of information on anaemia in the late postpartum period in Cameroon. Postnatal anaemia has devastating consequences. It increases the risk of postpartum depression, stress, and mental instability, as well as reduces mental performance [14]. It may also result in an insufficient milk production as such an impaired immunity for the child [15]. These can impair maternal care and interaction with the baby [14,16]. Endometritis has also been linked to anaemia [17].

The pregnancy-related mortality in Cameroon as of 2018 was 467 deaths per 100000 live births [18], which is 6.6 times higher than the expected value by 2030 as per the strategies for ending preventable maternal mortality (EPMM strategies) for achieving the third Sustainable Development Goals (SDGs) [19]. The annual change of this ratio from 2013 to 2017 ranges from 0.53% to 2.89% [20]. Anaemia is the direct cause of maternal mortality in deliveries complicated by postpartum haemorrhage, and it is also the leading indirect cause of pregnancy-related mortality in Cameroon [21]. The risk is higher in sub-Saharan Africa when compared to those in Europe and America, probably because of the poverty, malaria, and inadequate nutrition [22,23].

**Rationale:** anaemia in the LPNP is not routinely investigated. As such, when undiagnosed and untreated can progress for months, impairing the quality of life of the woman [14] and can progress into the subsequent pregnancy [24] as well as result in maternal death.

**Objectives:** to determine the prevalence and the severity of late postnatal anemia as well as its predisposing factors.

## Methods

**Study design:** a hospital-based cross-sectional study was conducted.

**Study setting:** the study was carried out from the 18^th^ February 2020 to 31^st^ of April 2020 at the Infant Welfare Clinics (IWC) of the Buea and Limbe Regional Hospitals, Cameroon. Both hospitals are secondary referral hospitals with antenatal clinics, maternity, theatres, midwives, general practitioners, as well as Obstetricians and Gynecologists. However, these settings lack a dedicated postpartum clinic or a system of community midwives to screen for LPNA.

**Study population:** the study population was composed of postpartum women who presented for the vaccination of their babies at the IWC of the BRH and LRH.

**Participants:** we included women between the 6^th^ and 27^th^ week postpartum who consented and those below 21 years for whom assents were obtained. Participants were selected via the consecutive sampling method.

**Variables:** our outcome variable was anaemia in the late postnatal period, defined as a capillary haemoglobin (Hb) less than 12g/dl between the 6^th^ to 27^th^ week postpartum. It was further classified into mild, moderate, and severe as an Hb 11.0-11.9g/dl, 8.0-10.9 g/dl and <8.0 g/dl respectively. Our predictor variables were subdivided into sociodemographic (age, marital status, highest level of education, employment status, religion and region of origin), antenatal and intrapartum (trimester of commencing antenatal clinic, the number of clinics attended, prenatal anemia, mode of delivery, intrapartum bleeding and birth weight of neonate) and the postpartum variables (post-partum transfusion, postpartum hematinic prescription, adherence to the hematinic, lactation, onset of menstrual flow and mucocutaneous pallor).

**Data sources/measurement:** the predictor variables were obtained from a face-to-face interview using a questionnaire that was designed and piloted. It had subsections on sociodemographic, medical, obstetric, antenatal care, delivery, and postnatal history, as well as examination for mucocutaneous pallor. The capillary haemoglogin was measured using the Sejoy® Haemoglobinometer. The third or fourth finger of the non-dominant hand was swabbed twice with 70% alcohol, and then a drop of capillary blood was obtained on the strip. The strip was then inserted into the haemoglobinometer for Hb measurements.

**Bias:** recall bias was one of the limitations we predicted; as such, we narrowed down the questions in the questionnaire to things that could easily be remembered by our participants.

**Study size:** the study size was calculated using Cochrane´s formula with a prevalence (p) of 64.4% obtained from a study which was carried out on postpartum anaemia amongst women between 6 weeks and 12 months in Uganda [25], giving a minimum sample size of 353 participants.

**Quantitative variables:** the quantitative variables were categorized in frequencies. We also calculated the means and medians.

**Statistical methods:** data collected was coded to ensure confidentiality, cross-checked, and entered into SPSS Version 23. The prevalence of anaemia was calculated as the proportion of women with a haemoglobin level less than 12g/dl between the 6^th^ to 27^th^ week postpartum, concerning the total population of women studied. The severity of anaemia for the population was categorized into mild, moderate, and severe based on the proportion of the population with these haemoglobin ranges: 11-11.9g/dl, 8.0-10.9 g/dl, and <8.0 g/dl, respectively. The relationship between LPNA and predictor variables was analysed by binary logistic regression for odds ratios and p-values after categorisation of predictor variables. A P value of < 0.05 at a 95% Confidence Interval was our cutoff for statistical significance and independent association.

**Ethical approval:** it was obtained from the Faculty of Health Sciences Institutional Review Board - University of Buea. Approval was then obtained from the Buea and Limbe Regional hospitals with ethical approval numbers 20333/2020 and 364/MPH/SWR/RHL/DO, respectively.

## Results

**Figure 1:**
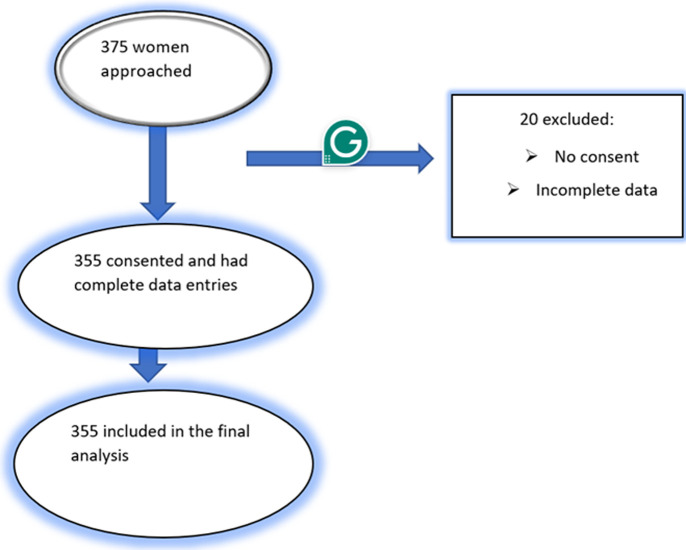
flow diagram for participant inclusion

A total of 375 women were approached, but only 355 were included in our final analysis because they consented to the study and had complete data entries (Figure 1).

**Descriptive statistics:** the age range of the participants was from 16 to 42 years. The mean age was 27.6±5.3, with most of the women being between 21 and 35 years. About half of the women had a post-secondary level education as their highest level of education. Sixty-three-point four percent (63.4%) of the women were married. All but one of the participants were Christians. Fifty-eight percent (58%) of the participants were employed. A majority of the women originated from the North West Region of Cameroon (Table 1). Comorbidities were reported amongst 25 (7%) participants. Of these, 60% were People Living with HIV. The remainder had hepatitis B 3 (12%), hypertension 3 (12%), peptic ulcer disease 3 (12%) and diabetes 1 (4%).

**Table 1:** the sociodemographic characteristics of the study population

Variable	Category	Frequency (%)
Age	16-20	35(9.9)
	21-35	289(81.4)
	≥36	31(8.7)
Educational status	None	2(0.6)
	Primary	27(7.6)
	Secondary	143(40.3)
	Post-secondary	183(51.5)
Marital status	Single	130(36.6)
	Married	225(63.4)
Employment status	Employed	206(58)
	Unemployed	149(42)
Religious affiliation	Christian	354(99.7)
	Muslim	1(0.3)
Region of origin	Center	4(1.1)
	Littoral	8(2.2)
	South west	137(38.5)
	North west	161(45.3)
	West	36(10.1)
	North	5(1.4)
	Others	4(1.1)

**Figure 2:**
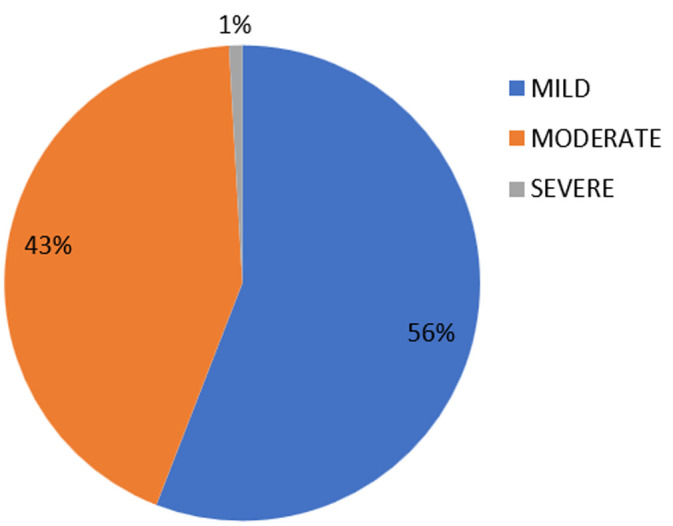
severity of late postnatal anaemia

**Outcome data:** out of the 355 participants, 109 had a haemoglobin below 12g/dl, giving a prevalence of 30.7 %. Of these, 61 (56%) had mild LPNA, 47 (43.3%) had moderate LPNA whereas only 1 (0.9%) had severe LPNA (Figure 2).

**Main results:** upon using the binary logistic regression model for associations, we found a low level of education, commencing antenatal care in the 3^rd^ trimester, prenatal anaemia, a low baby´s birth weight, and mucocutaneous pallor to be statistically significantly associated with LPNA (Table 2, Table 3 and Table 4).

**Table 2:** association between late postnatal anaemia and sociodemographic characteristics

Variable	Anaemia; yes	No	Total	OR	95%; lower	CI; upper	P-value
**Age**							
≥36	13(41.9)	18(58.1)	31	1.031	0.356	2.986	0.955
21-35	85(29.4)	204(70.6)	289	0.610	0.284	1.310	0.205
16-20	16(47.5)	19(54.3)	35	1			
**Marital status**							
Single	50(38.5)	80(61.5)	130	1.549	0.960	2.501	0.073
Married	64(28.4)	161(71.6)	225	1			
**Highest level of education**							
Primary	14(48.1)	15(51.9)	29	2.476	1.094	5.605	**0.030**
Secondary	51(35.7)	92(64.3)	143	1.418	0.866	2.321	0.165
Post-secondary	49(26.8)	134(73.2)	183	1			
**Employment status**							
Employed	66(32.0)	140(68.0)	206	0.966	0.592	1.577	0.890
Unemployed	48(32.2)	101(67.8)	149	1			

CI: confidence interval; OR: odd ratio

**Table 3:** association between Late postnatal anaemia and antenatal and intrapartum characteristics

Variable	Anaemia; yes	No	Total	OR	95%; lower	CI; upper	P-value
**Trimester of starting ANC, n=353**							
1^st^	38(22.9)	129(77.1)	167	0.243	0.109	0.540	0.001
2^nd^	59(38.1)	96(61.9)	155	0.508	0.233	1.111	0.090
3^rd^	17(54.8)	14(45.2)	31	1			
**Number of ANC consultations**							
1	3(100)	00	3	1.441	0.124	16.782	0.771
2-4	49(40.8)	71(59.2)	120	1.151	0.711	1.863	0.568
>4	61(26.5)	169(73.5)	230	1			
**Prenatal anaemia**							
No	73(27.7)	191(72.3)	264	0.453	0.272	0.753	0.002
Yes	38(45.8)	45(54.2)	83	1			
**Mode of delivery**							
Vaginal	85(32.1)	180(67.9)	265	1.103	0.545	2.231	0.786
Elective CS	7(24.1)	22(75.9)	29	0.587	0.211	1.630	0.307
Emergency CS	21(34.4)	22(75.9)	61	1			
**Intrapartum bleeding**							
Mild	79(30.9)	177(69.1)	256	1.102	0.338	3.593	0.872
Moderate	12(31.6)	26(68.4)	38	0.690	0.277	1.719	0.426
Severe	9(39.1)	14(60.9)	23	0.638	0.208	1.958	0.432
Did not know	14(36.8)	24(63.2)	38	1			
**Birth weight of the neonate**							
≤2.5	12(44.4)	15(55.6)	27	2.801	1.016	7.722	0.047
2.6-3.9	94(32.1)	199(67.9)	293	1.665	0.813	3.410	0.163
≥4.0	8(22.9)	27(77.1)	35	1			

ANC: antenatal care; CI: confidence interval; CS: cesarean section

**Table 4:** association between late postnatal anaemia and postpartum characteristics

Variable	Anaemia; yes	No	Total	OR	95%; lower	CI; upper	P-value
**Post-partum transfusion**							
No	109(31.6)	236(68.4)	345	0.357	0.074	1.731	0.201
Yes	5(50.0)	5(50.0)	10	1			
**Postpartum haematinic prescription n=351**							
No	40(41.2)	57(58.8)	97	0.857	0.144	5.093	0.866
Yes	72(28.3)	182(71.7)	254	1			
**Haematinic adherence, n=260**							
No	23(24.5)	71(75.5)	94	0.857	0.376	1.264	0.229
Yes	51(31.9)	109(58.1)	160	1			
**Lactation**							
No	3(37.5)	5(62.5)	8	2.694	0.454	15.968	0.275
Breastfeeding only	72(32.9)	147(67.1)	219	1.158	0.628	2.134	0.639
Mixed feeding	39(30.5)	89(69.5)	128	1			
**Onset of menses**							
No	36(31.0)	80(69.0)	116	1.500	0.794	2.833	0.211
Yes	78(32.8)	160(67.2)	238	1			
**Mucocutaneous pallor**							
No	83(27.5)	219(72.5)	302	0.255	0.122	0.533	<0.001
Yes	31(58.5)	22(41.5)	53	1			

CI: confidence interval; OR: odd ratio

## Discussion

There were 109 women out of the 355 participants with anemia, giving a prevalence of 30.7%. A majority of whom had mild to moderate anemia, 61 (56%) and 46 (43.1%) respectively, with only 1 (0.9%) participant with severe anemia. The highest level of education (p= 0.030), trimester of commencing antenatal care consultations (p= 0.001), prenatal anemia (p= 0.003), the baby´s birth weight (p= 0.047), and mucocutaneous pallor (p<0.001) were significantly and independently associated with late postnatal anemia. We had a limitation of recall bias. This is because most of our participants did not have documented records of their past pregnancy and delivery. As such, they only answered some of the questions based on what they could remember.

**Figure 3:**
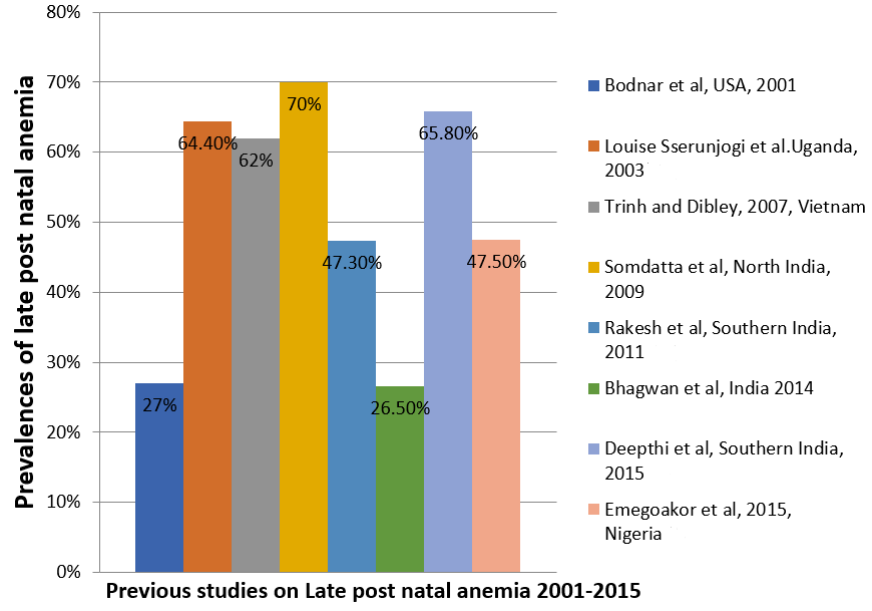
trends of the prevalence of late postnatal anaemia from 2001 to 2015

**The prevalence of late postnatal anaemia:** the prevalence of LPNA was 30.7%. This finding was similar to the 26.5% and 27% reported by Bodnar *et al*. and Bhagwan *et al*. obtained in Coastal Karnataka in 2014 and the USA in 2001, respectively [26,27]. The similarity with that of Bhagwan *et al*. could be related to the fact that their study site had better health facilities, higher literacy rates, and universal antenatal coverage as well as more institutional deliveries [27], which was also the case with our study setting. Though 27% was obtained in the USA following a study amongst low-income women in 2001 [26], it only implies that we are about 2 decades behind in our struggle against LPNA. Figure 2 demonstrates the trends of LPNA from 2001 to 2015, and Table 5 shows a summary of the various studies conducted, prevalence, and risk factors on LPNA between 2001 and 2015 (Figure 3).

**Table 5:** previous studies on postnatal anaemia, prevalence, and statistically significant factors

Author/country/year	Study design/sample size	Prevalence of LPNA	Statistically significant factors
Bodnar *et al*. USA, 2001	Retrospective Cohort Analysis - 59,428 participants between 4 weeks and 26 weeks postpartum	27% (Anaemia = Hb < 12.0 g/dl for women at least age 15 years)	Prenatal anaemia; maternal obesity; multiple birth; and not breastfeeding
Louise Sserunjogi *et al*. Uganda, 2003	Cross-sectional study -349 mothers between 6 weeks and 12 months	64.4% (Anaemia = Hb <12g/dl)	Iron supplementation; excessive bleeding during or after delivery
Trinh and Dibley, 2007, Vietnam	Cross-sectional survey -348 women between 0 and to 6months postpartum	62% (Anaemia = Hb < 12g/dl)	Women aged 30 years or older
Somdatta *et al*. North India, 2009	Prospective community-based study -168 women at 6 weeks postpartum	70% (Anaemia = Hb<11g/dl)	Consumption of 90 IFA tablets
Rakesh *et al*. Southern India, 2011	Prospective Cohort study; 93 women at 36th to 38th week of pregnancy and the 2nd and 6th week postpartum.	47.3% (Anaemia = Hb<12g/dl)	Anaemia in the third trimester of pregnancy; heavy bleeding was perceived by the mother during delivery; younger maternal age; inadequate iron supplementation during the postpartum period
Bhagwan *et al*. India 2014	Community-based cross-sectional study; 401 women between 6 to 14 weeks postpartum.	26.5% (Anaemia = Hb<12gm/dl)	Illiteracy
Deepthi *et al*. Southern India, 2015	Cross-sectional Study. - 647 women between 0 to 23 months postpartum	65.8% (Anaemia = Hb<12g/dl)	Educational status of the woman; consumption of iron tablets during pregnancy.
Emegoakor *et al*. 2015, Nigeria	Prospective longitudinal study; 202 women with uncomplicated singleton pregnancies from term to 6 weeks postpartum	47.5% (Anaemia = Hb< 11g/dl)	Employment status; anaemia at term; anaemia at 48 h postpartum; low ferritin concentration at 48 h postpartum.

**Severity of late postnatal anemia:** with regards to the severity of LPNA, our findings were similar to those of Sserunjogi *et al*. and Bhagwan *et al*. who had more women with mild and moderate anaemia and few or none with severe anaemia [25,27]. Severe anaemia usually presents with frank symptoms of anaemia, like increased frequency of tiredness, breathlessness, palpitations, which makes early diagnosis and treatment more probable than asymptomatic forms of anaemia [17,28]. Most women with severe anaemia must have consulted and had been managed before our contact with them at the IWC. Also, the further away the woman is from delivery, there is the expectation that anaemia related to pregnancy resolves, so that more severe anaemia is found earlier on in the PNP.

**Factors predisposing to late postnatal anemia:** anemia occurring in the postnatal period, be it in the acute, early, or late phase, is caused by pre-partum iron deficiency anaemia and intra-partum hemorrhage [2,13,29]. However, our study sought to find out the factors that can predispose to anaemia in the late postnatal period. Women who had a primary school education as their highest level of education had a higher prevalence and odds of LPNA when compared with those who had secondary and post-secondary education. This finding was similar to that of Bhagwan *et al*. [27]. This finding could be explained by the fact that the level of education is directly related to health seeking behaviour and adherence to medical treatment as well as socio-economic power to purchase treatment of anaemia These women probably did not read more about anaemia in pregnancy and in the postpartum from sources like the internet when compared to their counterparts.

Participants who started antenatal care (ANC) in the 3^rd^ trimester had the highest prevalence of LPNA, closely followed by those who started in the 2^nd^ and 1^st^ trimesters (Table 3). This finding was similar to that obtained by Bodnar *et al*. [26]. This could be explained by the fact that they probably consume less than 90 Iron and Folic acid tablets because of the late start of their ANC consultations [5,30]. This could also be related to the fact that these women probably missed the prophylactic treatment against Malaria and Hookworm infestations, respectively, which are some causes of anaemia in pregnancy [31]. These women probably attended very few lessons, and as such did not know or understand the importance of haematinic during and after pregnancy. Most importantly, they had limited time for their anaemia to be corrected, and this process of correction can persist well into and after the puerperium. More women who had anaemia in their last consultation of ANC were found to be anaemic in the late postpartum. This was similar to what Bodnar *et al*. found, that is, prenatal anaemia as the main predictor for postnatal anaemia [26]. These findings were following what Milman documented in his review on postpartum anaemia, which states that prenatal anaemia is one of two main causes of postnatal anaemia [2]. This could be explained by the fact that most of these women had uncorrected prenatal anaemia, which was aggravated by intrapartum haemorrhage. Consequently, a progression of anaemia into the postpartum period due to the limited iron reserves by the 6^th^ week postpartum [32].

Mothers with babies who weighed less than 2.5kg had a higher prevalence of anaemia when compared to their counterparts. This is because most of these ladies probably had premature babies or babies with intrauterine growth restriction who needed special care; as such, these mothers probably focused on caring for their babies and thereby reducing the chances of consuming hematinic in the postnatal period, the reason for the anaemia. This cohort of women may also carry other comorbidities or medical conditions, which explains their fetal IUGR. Amongst our participants, the likelihood of having a mucocutaneous pallor and being anaemic was high. This finding was similar to that of Sserungoji *et al*. [25]. This could be explained by the fact that one of the cardinal signs of anaemia is pallor of mucocutaneous surfaces like the conjunctivae, tongue, palmar and plantar surfaces, which usually requires laboratory testing for confirmation [33]. Therefore, its assessment should not be neglected amongst postpartum women.

## Conclusion

Though often neglected, late postnatal anaemia is relatively common in this study setting, demonstrating a prevalence of 3 in 10 women affected. However, most cases are mild to moderate anemia. Determinants were a low level of education, late commencement of antenatal care, prenatal anemia, low birth weight babies, and mucocutaneous palor. The preventive measures should therefore include: formal education for the girl child beyond the primary level, commencing antenatal care in the first trimester, managing prenatal anemia properly, ensuring mothers with low birth weight babies receive hematinic and anaemia in the late postnatal period.

### What is known about this topic

- The prevalence of anaemia in the postnatal period in some upper-middle- and high-income countries like the United States of America, India, Vietnam, Uganda, and Nigeria;
- Some factors associated with the postnatal anaemia include age, educational level, prenatal anaemia, maternal obesity, multiple birth, lactation, iron supplementation, and excessive bleeding during or after delivery.

### What this study adds

- The prevalence of late postnatal anaemia in Cameroon, one of the lower-middle-income countries which should raise a concern about the late postnatal follow-up for women in such countries;
- The commencement of antenatal care in the third trimester of pregnancy was found as a precipitating factor for LPNA; as such, a call for women of childbearing age should be encouraged to commence antenatal care as soon as pregnancy is diagnosed;
- The presence of mucocutaneous palor as a precipitating factor for LPNA is a wake-up call for physicians and nurses attending to women in the LPNP to thoroughly examine mucocutaneous membranes for palor.
